# Abdominal cocoon accompanied by multiple peritoneal loose body

**DOI:** 10.1097/MD.0000000000006185

**Published:** 2017-03-03

**Authors:** Yongyuan Cheng, Lintao Qu, Jun Li, Bin Wang, Junzu Geng, Dong Xing

**Affiliations:** a Department of Radiology, Yantai Municipal Laiyang Central Hospital; b Medical Imaging Research Institute, Binzhou Medical University; c Department of Radiology, Yantai Yuhuangding Hospital, Medical College of Qingdao University, Yantai, Shandong, China.

**Keywords:** abdominal cocoon, CT, magnetic resonance imaging, peritoneal loose body

## Abstract

**Rationale:**

Abdominal cocoon and peritoneal loose body are both rare abdominal diseases.

**Patient concerns:**

The patient reported in this case was a 47-year-old man who suffered from abdominal pain and distension for 3 days.

**Diagnosis:**

X-ray, computed tomography, and magnetic resonance imaging revealed multiple peritoneal loose body and small bowel obstruction, characterized by a total encapsulation of the small bowel with a fibrous membrane.

**Interventions:**

The patient underwent surgical treatment and exploratory laparotomy confirmed the diagnosis of abdominal cocoon.

**Outcomes:**

Histopathological examination of pelvic nodules confirmed peritoneal loose body.

**Lessons:**

To our knowledge, the herein reported case is the first abdominal cocoon that was accompanied by multiple peritoneal loose body.

## 1 Introduction

Abdominal cocoon (AC) is a rare clinical abdominal syndrome characterized by cocoon-like intestine due to all or part of the small intestine, which is covered by a layer of dense, gray, and white fibrous membrane. The preoperative imaging diagnosis was difficult.^[1–4]^ Peritoneal loose body (PLB) is another rare abdominal disease, which lacks the understanding of its imaging manifestations.^[5]^ Clinical symptoms of AC were often nonspecific, and PLB rarely resulted in clinical symptoms. In the previous literature, there was no report of AC combined with PLB. Herein, we report the X-ray, computed tomography (CT), and magnetic resonance imaging (MRI) findings of a case of AC accompanied by multiple PLB.

## 2 Case report

A 47-year-old man suffered from abdominal pain and distension, accompanied by nausea and vomiting for 3 days. The patient had a history of frequent abdominal distension. He had no history of trauma or surgery. A local abdomen examination revealed a large lump in the middle lower abdomen with no obvious tenderness. The boundary of the lump was not clear but soft. The bowel sound was normal. Routine laboratory studies demonstrated no abnormalities.

**Figure 1:**
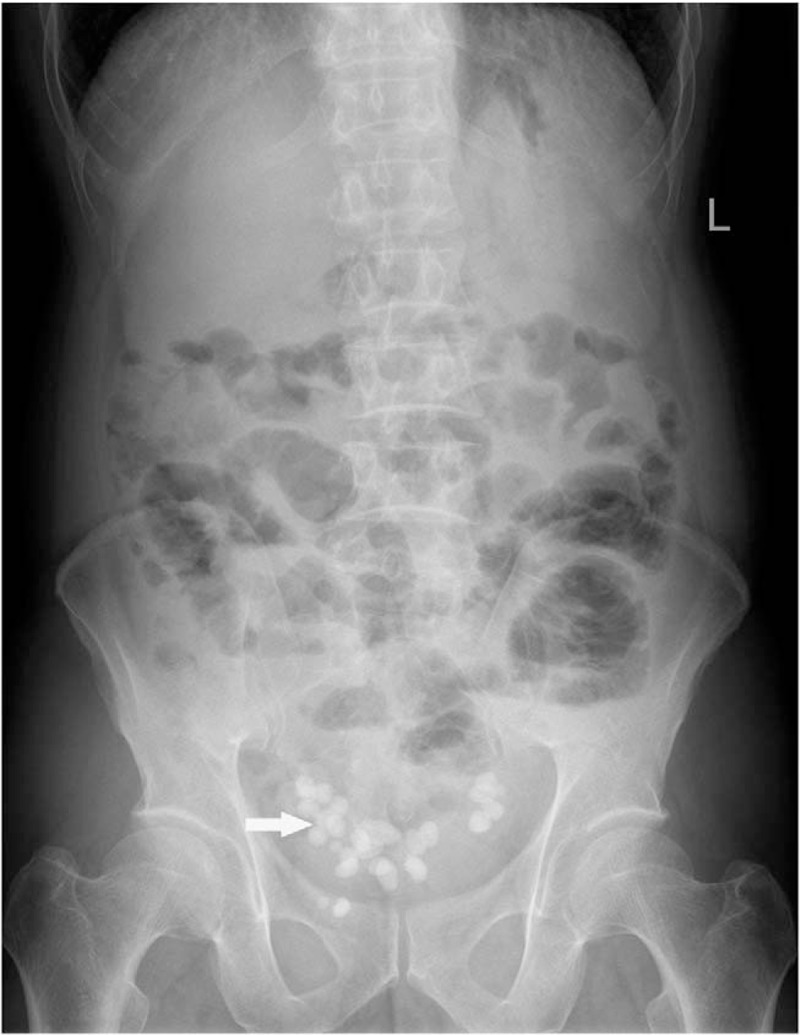
X-ray plain abdominal imaging shows dilatation and air-fluid level within intestine, and multiple high-density nodules with clear boundary in pelvic cavity (arrow).

**Figure 2:**
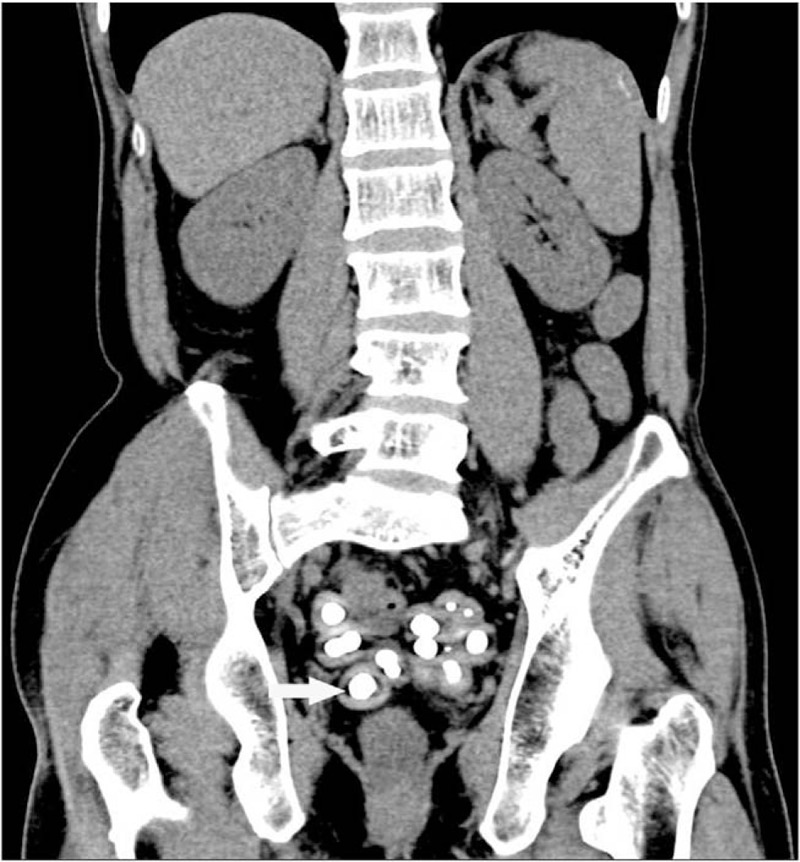
Abdominal coronal and sagittal CT images show multiple nodules in pelvic cavity, with central calcification and peripheral ring-like equidensite (arrow). Sagittal CT image shows dilated and tortuous intestine. CT = computed tomography.

**Figure 3:**
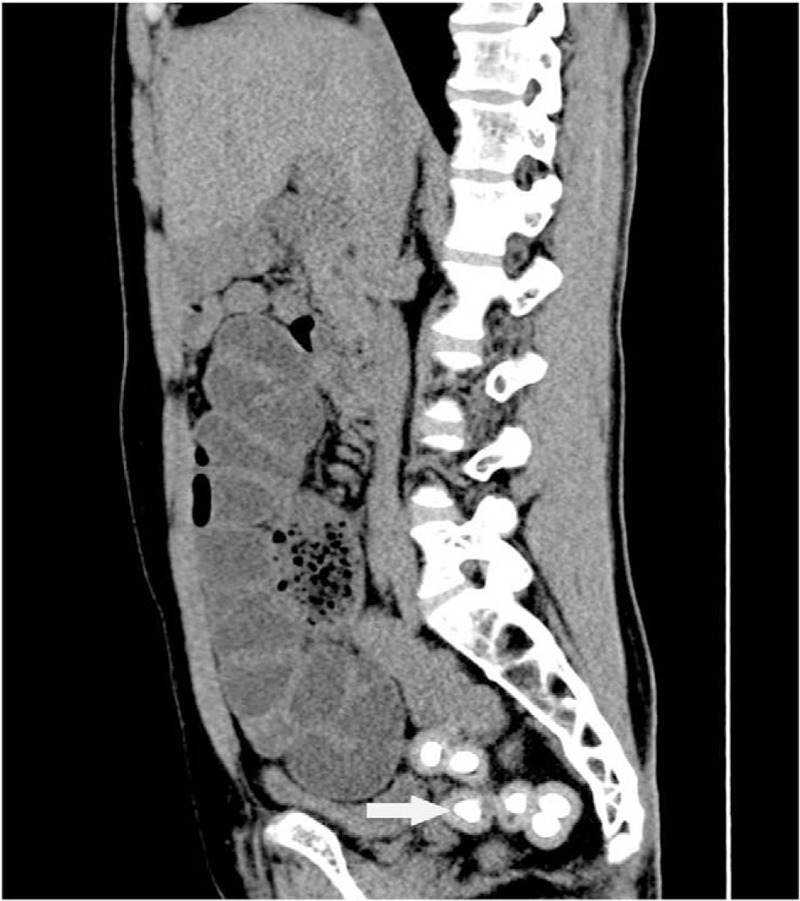
Abdominal coronal and sagittal CT images show multiple nodules in pelvic cavity, with central calcification and peripheral ring-like equidensite (arrow). Sagittal CT image shows dilated and tortuous intestine. CT = computed tomography.

**Figure 4:**
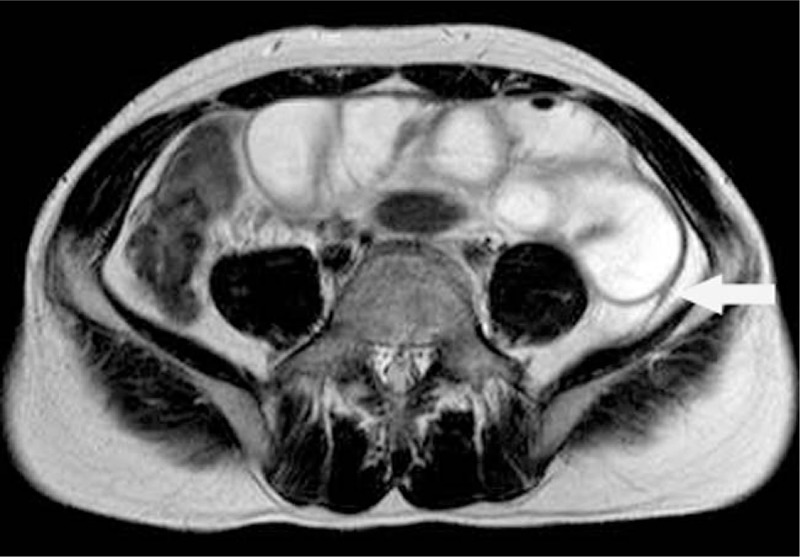
Abdominal axial T2WI MR images show dilated and tortuous intestine, with linear low signal encapsulated (arrow). MR = magnetic resonance.

**Figure 5:**
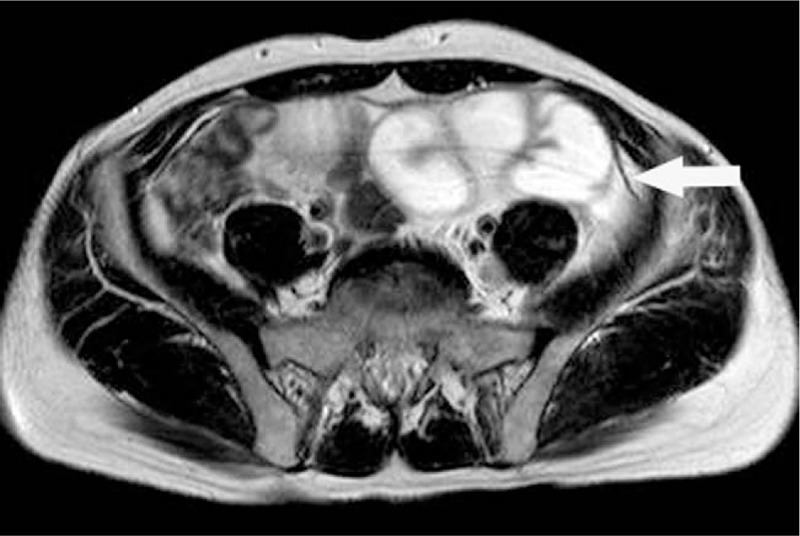
Abdominal axial T2WI MR images show dilated and tortuous intestine, with linear low signal encapsulated (arrow). MR = magnetic resonance.

**Figure 6:**
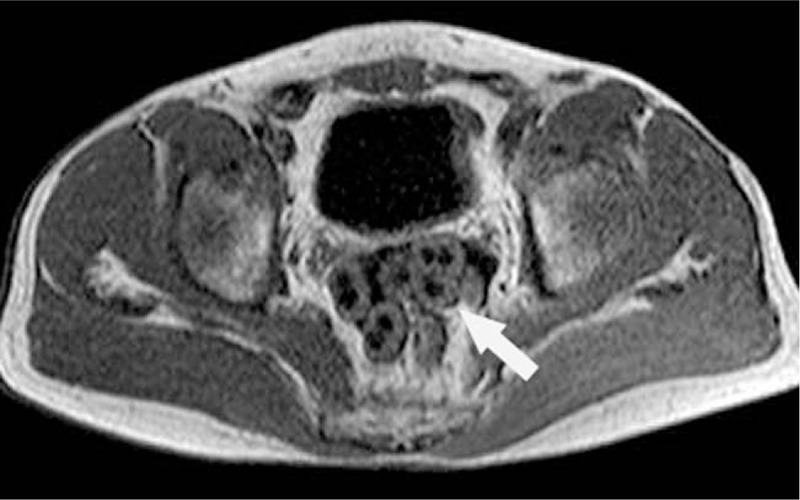
MR images show multiple abnormal nodules in rectovesical pouch. T1WI reveals central hypointensity and peripheral ring-like isointensity (arrow), T2WI reveals homogeneously hypointensity (arrow). MR = magnetic resonance.

**Figure 7:**
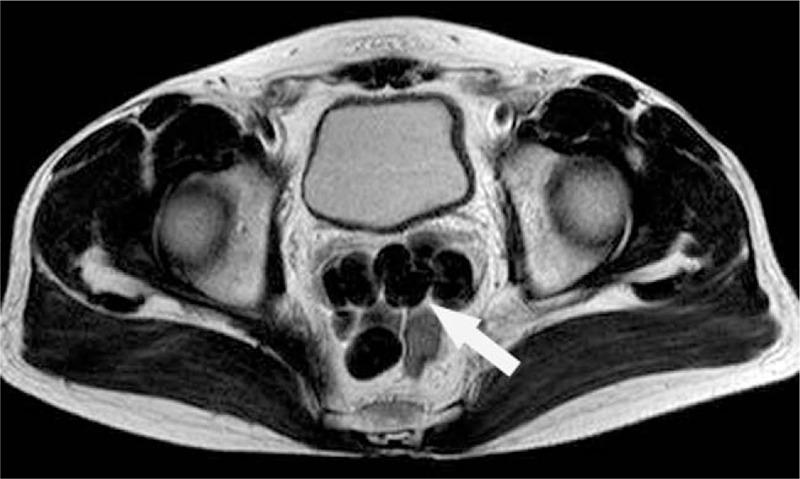
MR images show multiple abnormal nodules in rectovesical pouch. T1WI reveals central hypointensity and peripheral ring-like isointensity (arrow), T2WI reveals homogeneously hypointensity (arrow). MR = magnetic resonance.

This study was approved by Ethic Committee of Binzhou Medical University and the patient provided the written informed consent to participate in this study. X-ray of the abdomen showed small bowel dilatation in the middle lower abdomen with several step-ladder like air-fluid levels and a number of bead-like high-density nodules with clear boundary in pelvic cavity (Fig. 1). CT scan demonstrated partial small intestine gathering with dilatation and fluid filling in the middle and lower abdomen. And a number of high-density nodules with clear boundary were found in pelvic cavity. These nodules were unequal in size and surrounded by equi-density rings (Figs. 2 and 3). MR examination also revealed dilatation and fluid filling in small intestine in the middle and lower abdomen. TSE-T2WI images showed linearly low signal encapsulating the dilated intestine (Figs. 4 and 5). A number of abnormal signal nodules were scattered in the pelvic cavity, with low signal intensity in the middle and peripheral ring-like iso-intensity signal on T1WI image, and with circular or elliptical low signal on T2WI image (Figs. 6 and 7). Small bowel obstruction combined with multiple peritoneal loose body was preoperatively diagnosed by these imaging features.

**Figure 8:**
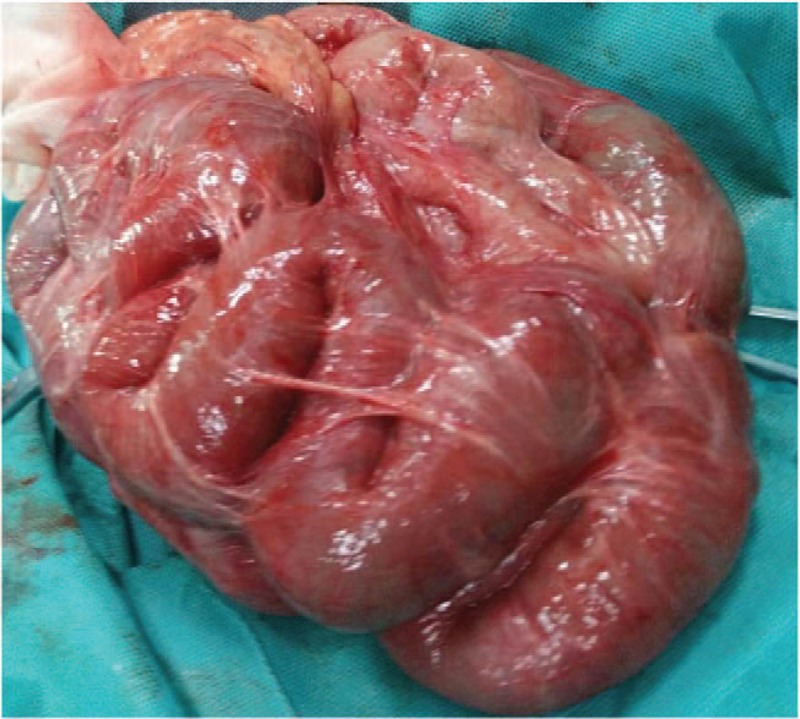
Intraoperative photograph shows the encapsulated small bowel segments with a dense fibrous layer.

**Figure 9:**
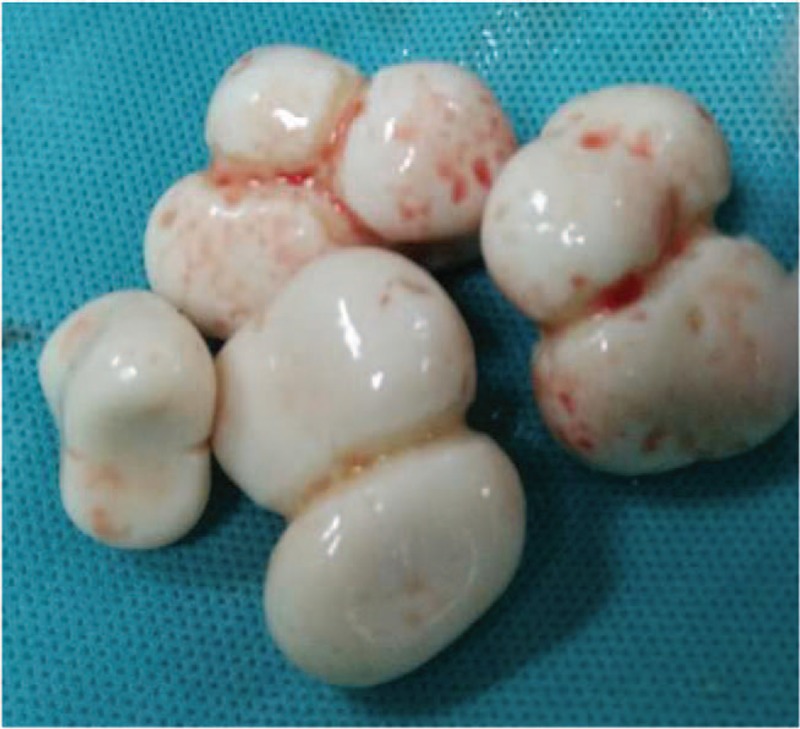
Operation finding shows multiple gray and white oval nodules with smooth surface in pelvic cavity.

**Figure 10:**
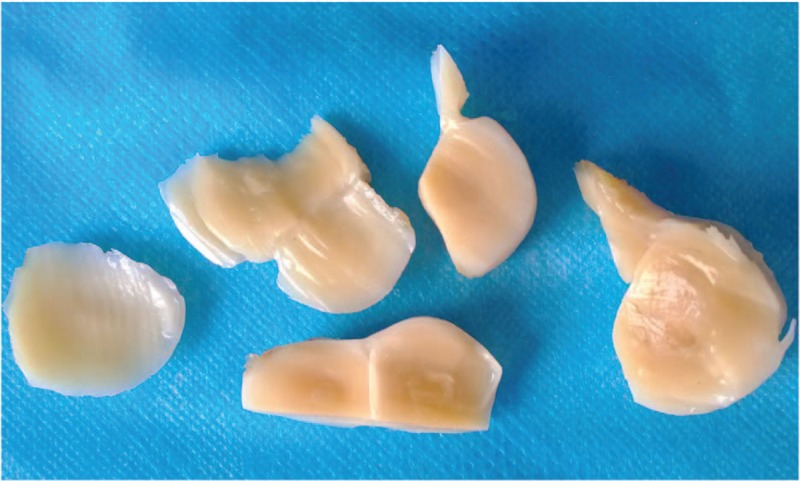
Gross anatomy of gray and white nodules shows sallow section, with central hard nodules.

**Figure 11:**
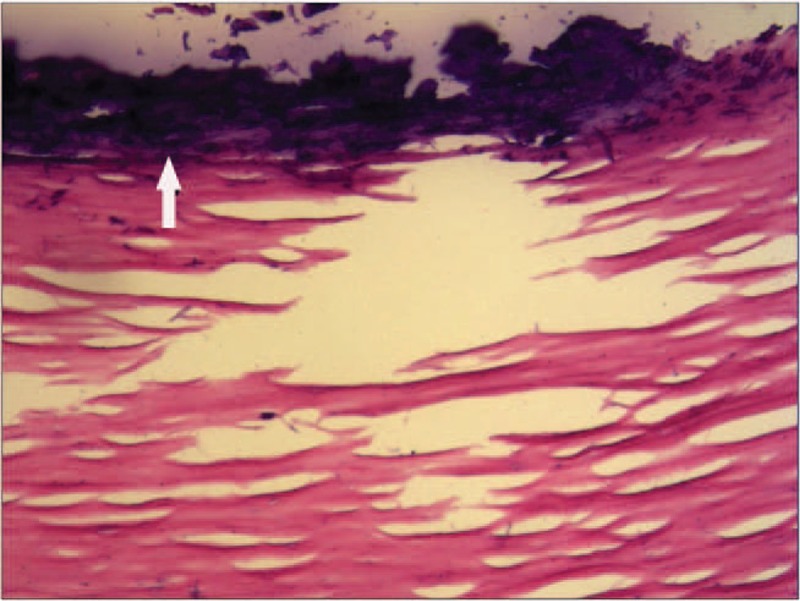
Hematoxylin–eosin staining (original magnification ×10). Histological examination confirms the diagnosis of peritoneal free body (arrow).

Exploratory laparotomy revealed extensive membranous adhesions in abdominal cavity. The entire small intestine was wrapped around with gray and white fibrous membrane. About 30 cm of the small intestine expanded significantly with thick contents at the right abdomen. Abdominal cocoon was diagnosed intraoperatively. Lysis of adhesions and intestinal arrangement were performed (Fig. 8). Multiple gray and white circular masses were found in the pelvic cavity, with size of about 2 to 3 cm, with free, hard and smooth surface, sallow section, and central hard nodules (Fig. 9). Under the microscope, the central yellow part was the necrotic adipose tissue. A circular hard area with the thickness of 0.5 cm around fat tissues was calcification. Peripheral substances like “cooked chicken protein” were homogeneous, red dye fibrous connective tissue with concentric lamellar arrangement (Figs. 10 and 11). The results of pathological examination were in accordance with the peritoneal loose body.

## 3 Discussion

Abdominal cocoon is also called sclerosing encapsulating peritonitis and is divided into primary and secondary types. Primary AC is a disease of uncertain origin. It may be due to poorly developed greater omentum in the embryonic stage which downs the colon transversum and encapsulate small intestine. Secondary AC often has multiple causes such as abdominal dialysis, long-term therapy of beta blocker use, abdominal tuberculosis, peritonitis, liver cirrhosis, abdominal surgery history, organ transplantation, systemic lupus erythematosus, and gastrointestinal malignancies. All of these ingredients can stimulate peritonitis and lead to a large amount of fibrin in the abdominal cavity. Due to absorption disorders and connective tissue proliferation, a layer of dense fibrosis capsule may be formed to encapsulate the small intestine eventually.^[1–4]^

The accurate cause of peritoneal loose body remains unknown. The main sources include appendices epiploicae, intestinal fat, greater retinal fat, fat deposits of lymph nodes, fat in the pancreas, etc. Free floating loose bodies are formed due to ischemia, necrosis, and exfoliation of adipose tissue in these sites. Then internal calcifications occur gradually, and their volumes snowball slowly due to continuous absorption of the protein in the ascites. Pathological observations revealed a calcified layer in the center of the free body around fat. Peripheral substances like “cooked chicken proteins” were homogeneous, red dye fibrous connective tissue with concentric lamellar arrangement, supporting this hypothesis.^[5–7]^

AC is lack of specific clinical manifestations, and the initial clinical symptoms are intestinal obstruction or/and abdominal mass. AC may recurrently attack, be subsided, or increasingly get severe. The shape and size of most intraperitoneal free bodies are mainly round or oval mass with 0.5 to 2.5 cm in diameter, similar to this case. These PLB rarely cause symptoms. However, when the size of PLB in diameter increases from 5 to 9.5 cm, pelvic organs can be compressed due to the limited space of pelvis. Sometimes, incomplete intestinal obstruction and the symptoms of frequent urination may occur.^[5–7]^

AC can be divided into localized and diffuse types according to the scope of the encapsulated small intestine. The former is partial involvement of small bowel and the latter is total involvement of small bowel. The characteristic imaging findings of AC are envelope sign, defined as the small intestine surrounding by a layer of thick membranous structure. The small intestine surrounded by fibrous membrane is disorder, tortuosity, aggregation, and fixation. The fibrous membrane may be enhanced by contrast enhancement scanning. In this study, it was easy to be misdiagnosed for the difficulty to distinguish the envelope sign due to extensive adhesion between fibrous membrane and intestinal wall. In combination with a literature review,^[1,2,8]^ enveloped sign is of great value in the diagnosis of the disease. When small bowel obstruction occurs with no clear cause, it should be carefully observed whether the small intestine could be encapsulated by fibrous membrane, especially in the liquid accumulation area or fat accumulation zone outside the intestine.

The most common imaging manifestation of PLB is the oval mass with central calcification and low-density area. Plain abdominal radiograph shows round or oval calcified mass in the pelvic cavity, which can move freely. CT is an important examination method for preoperative evaluation and differential diagnosis of PLB. CT examination showed concentric circle or oval mass with central calcification and peripheral soft tissue density, and often the mass would have a distinct fat plane separating it from adjacent organs.^[9]^ MR examination revealed circular low signal in the center and annular iso-intensity in the periphery on T1WI and uniform circular or oval low signal on T2WI. PLB should be differentiated as followings: calcification of the aneurysm, calcified lymph nodes, urinary calculi, fecal stones, and uterine fibroids calcification and other pelvic high-density nodules. Free mobility is an important feature of PLB in the diagnosis of other pelvic high-density nodules. In addition, no enhancement is helpful for the identification of PLB.^[5–7]^

AC and PLB are both rare clinical diseases. In this case, the possible relationship of AC and multiple PLB is ischemia, necrosis, and abscission of some greater omentum adipose tissue in the forming process of intestinal fibrous capsule. On the other hand, multiple factors of AC stimulate peritonitis and prompt a large number of fibrin deposition, leading to the formation of PLB.

Imaging findings and related clinical symptoms of AC and PLB should be understood and recognized by radiologists and clinicians to establish the correct diagnosis and treatment plan.
